# Books Reviewed

**Published:** 1863-08

**Authors:** 


### BOOKS REVIEWED.

*The Medical Student's Vade Mecum; a Compendium of Anatomy, Physiology, Chemistry, Poisons, Materia Medica, Pharmacy, Surgery, Obstetrics, Practice of Medicine, Diseases of the Skin, etc., etc. By* GEORGE MENDENHALL, M. D., *Professor of Obstetrics and Diseases of Women and Children in the Medical College of Ohio, Member of the American Medical Association, etc., etc. Seventh Edition, revised and greatly enlarged, with two hundred and twenty-four illustrations. Philadelphia:* LINDSAY & BLAKISTONE, 1863.

This is a very attractive and instructive book for young men pursuing the study of medicine, and while attending lectures it is invaluable. It furnishes a short and plain view of the most important facts and principles of the various sciences which engage his attention, and is arranged so as to refresh and more firmly fix upon his memory whatever is heard or read.— It is also very convenient for ready reference for the physician who may not have time to devote to reading more voluminous works. In cases of emergency when the young physician may be in doubt what course to pursue, it will prove a convenient and safe guide. It contains in short terms the principal facts of Anatomy, Physiology, Surgery, and Theory and Practice of Medicine and Materia Medica, and also a vast amount of information not included strictly under these heads. Its style is worthy of mention since it is so plain and so eminently within the comprehension of the young student. While we speak of it as especially adapted to the student, we by no means regard it as suited alone to students who have not yet received their diplomas, but physicians of experience will find it exceedingly valuable to them when they desire to refresh their memories with the anatomy, physiology or chemistry which they have forgotten.

This last edition is a great improvement upon the former ones, and as now presented to the profession it more than ever deserves its well merited popularity. It is truly astonishing to observe how many facts and how much direct teaching can be crowded into a small space. We most of all desire to call the attention of medical students to its merits, for there is no book better adapted for ready reference.

---

*A Manual of Minor Surgery. By* JOHN PACKARD, M. D., *Demonstrator of Anatomy in the University of Pennsylvania; one of the Visiting Surgeons to the West Philadelphia Military Hospital, etc., etc. With* 145 *illustrations. Authorized and adopted by the Surgeon-General of the United States Army for the use of Surgeons in the Field and General Hospitals.*

This is a convenient, well arranged, well written compendium of Minor Surgery, describing the instruments and materials used in dressing—application of dressings—modes of arresting hæmorrhage—sutures, etc., etc. It has chapters also upon Surgical Depletion—Anæsthesia—Bandages—Fractures—Dislocations—Cauterization and Injections—Foreign Bodies—Post Mortem Examinations—Disinfectants—Minor Surgical Operations. The Report of a Board, convened by order of the Surgeon-General, to examine the work with the view of its adoption for use in the Medical Department of the Army, declares that "it is satisfied that it is a better text-book upon the subject, than any of the treatises with which the American market has hitherto been supplied." This is saying a great deal where so many and so unobjectionable works have been published, and especially where so little differences exist, either in the material or manner of arranging it; they all contain the same facts; they all afford essentially the same teaching. This work pleases us very much, and its condensed, direct, plain style, greatly commends it to the profession.

*A Practical Hand-Book of Medical Chemistry. By* JOHN E. BOWMAN, F. C. S., *formerly Professor of Practical Chemistry in King's College, London. Edited by* CHARLES L. BLOXAM, *Professor of Practical Chemistry in King's College, London. Third American from the fourth and revised London edition, with illustrations. Philadelphia:* BLANCHARD & LEA, 1863.

Bowman's Medical Chemistry is the very book we have been wanting, for a long time. It contains in clear language and with accurate description the processes for examining the urine, bile, blood, milk, mucus, pus, etc, etc. Part V is upon the Detection of Poisons in Organic Mixtures—Arsenic, Antimony, Mercury, Lead, Copper, Zinc, Iodine, Sulphuric Acid, Hydrochloric Acid, Nitric Acid, Oxalic Acid, Hydrocyanic Acid, Opium, Strychnine, Nicotia, Phosphorus, Alcoloids, etc., etc. It is a valuable book for every physician, and there can be nothing better adapted to the wants of the medical student. It is indispensable to the physician in the rural districts where they are obliged to rely wholly upon themselves, and cannot at once refer their chemical examinations to professed chemists. In a word, it is a jewel of a book, and all physicians should buy it. In our miscellaneous department will be found a short extract from it upon the adulteration of milk. This is a specimen of the book, and will give an idea of its general style.

---

*A Practical Treatise on Fractures and Dislocations. By* FRANK HASTINGS HAMILTON, A. B., A. M., M. D., *Lt. Col. Medical Inspector U. S. A.; Professor of Military Surgery and Hygiene and of Fractures and Dislocations in Bellevue Hospital Medical College; one of the Surgeons to Bellevue Hospital, New York; Professor of Military Surgery, etc. in the Long Island College Hospital, Brooklyn; author of a Treatise on Military Surgery. Second edition, revised and improved. Illustrated with two hundred and twenty-five wood cuts. Philadelphia:* BLANCHARD & LEA, 1863.

We have very little occasion to speak of this book either by way of description or commendation. Our readers are for the most part familiar with the book, and well acquainted with its distinguished author. In the authorship of this book Dr. Hamilton has made for himself a world wide reputation, and at the same time he has accomplished more for the protection of Surgeons in legal proceedings than had ever been done before, and placed the profession under such lasting obligations that future generations of young surgeons will "rise up and call him blessed." Every form of fracture and dislocation is thoroughly described, together with the most proper mode of adjustment and retention. It is of untold value as a guide in practice, and at the same time it is a standard authority which will be everywhere recognized both by physicians and courts of justice. It has filled the hiatus which has long existed upon this subject, and taken its proper place with the most valuable of our standard Surgical works. The author tells us in his preface to this second edition that, "by a careful revision I have sought to render this edition, as far as possible, a faithful record of the progress of that branch of surgical science of which it treats. With this view some portions have been amended, some paragraphs have been excluded, and considerable additions have been made. The short chapter on "Gun-Shot Fractures" seemed to be demanded at this moment, and especially as the work has been placed upon the United States Army Supply Table for post and General Hospitals. Whether on the whole, by these new labors, the chaiacter of the volume has been improved, the reader must judge.

